# Applications of Nanomaterials in Electrochemical Enzyme Biosensors

**DOI:** 10.3390/s91108547

**Published:** 2009-10-27

**Authors:** Huihui Li, Songqin Liu, Zhihui Dai, Jianchun Bao, Xiaodi Yang

**Affiliations:** 1 Jiangsu Key Laboratory of Biofunctional Materials, College of Chemistry and Environmental Science, Nanjing Normal University, Nanjing 210097, China; 2 State Key Laboratory of Bioelectronics and Jiangsu Provincial Key Laboratory of Biomaterials and Biodevices, School of Chemistry and Chemical Engineering, Southeast University, Nanjing 210096, China

**Keywords:** electrochemical biosensor, nanomaterials, enzymes, review

## Abstract

A biosensor is defined as a kind of analytical device incorporating a biological material, a biologically derived material or a biomimic intimately associated with or integrated within a physicochemical transducer or transducing microsystem. Electrochemical biosensors incorporating enzymes with nanomaterials, which combine the recognition and catalytic properties of enzymes with the electronic properties of various nanomaterials, are new materials with synergistic properties originating from the components of the hybrid composites. Therefore, these systems have excellent prospects for interfacing biological recognition events through electronic signal transduction so as to design a new generation of bioelectronic devices with high sensitivity and stability. In this review, we describe approaches that involve nanomaterials in direct electrochemistry of redox proteins, especially our work on biosensor design immobilizing glucose oxidase (GOD), horseradish peroxidase (HRP), cytochrome P450 (CYP2B6), hemoglobin (Hb), glutamate dehydrogenase (GDH) and lactate dehydrogenase (LDH). The topics of the present review are the different functions of nanomaterials based on modification of electrode materials, as well as applications of electrochemical enzyme biosensors.

## 1. Introduction

A biosensor is defined as a type of analytical device incorporating a biological material, a biologically derived material or a biomimic intimately associated with or integrated within a physicochemical transducer or transducing microsystem [1]. Especially, there has been substantial progress in the past decade in electrochemical biosensors of biomolecules (especially enzymes). Nanomaterials with attractive electronic, optical, magnetic, thermal and catalytic properties have attracted great attention due to their widespread applications in physics, chemistry, biology, medicine, materials science and interdisciplinary fields. Recently, owing to their unique physical and chemical properties, nanomaterials are of considerable interest in the biosensor field, which have led to novel biosensors that have exhibited high sensitivity and stability [2-5]. Nanomaterials prepared from metals, semiconductors, carbon or polymeric species, shaped into nanoparticles and nanotubes, have been widely investigated for their ability as electrode modification materials to enhance the efficiencies of electrochemical biosensors. Besides, carbon nanofiber (CNF), a new nano-material used recently for oxidase substrates using dehydrogenase and oxidase, shows excellent catalytic activity [6-8].

Electrochemical biosensors incorporating enzymes with nanomaterials, which combine the recognition and catalytic properties of enzymes with the electronic properties of various nanomaterials, are new materials with synergistic properties originating from the components of the hybrid composites. Therefore, these systems have excellent prospects for interfacing biological recognition events with electronic signal transduction so as to design a new generation of bioelectronic devices with high sensitivity and stability.

In recent years we have seen an explosion in particularly useful applications of nanomaterials in electrochemical biosensors. Several comprehensive review articles have partially summarized recent advances in the field [9-19]. Wang *et al.* presented an overview of the synthesis and electrochemical applications of gold nanoparticles [9]. Suni *et al.* recently reviewed electrochemical sensors, which employ nanomaterials, concentrating mainly on gold nanoparticles and carbon nanotubes and which utilize only electrochemical impedance spectroscopy for analyte detection [17]. Tamiya *et al.* gave an overview on the application of nanomaterial-based biosensors, paying particular attention to gold nanoparticles and carbon nanotube-based label-free approaches [18]. Merkoci *et al.* described electrochemical analysis and biosensing using some common gold nanoparticles and quantum dots [19], but there has been no comprehensive overview on the application of nanomaterials in constructing diverse electrochemical sensors of various enzymes. In this review, we describe approaches that involve nanomaterials in direct electrochemistry of redox proteins, especially our own work on biosensor design immobilizing glucose oxidase (GOD), horseradish peroxidase (HRP), cytochrome P450 (CYP2B6), hemoglobin (Hb) and lactate dehydrogenase (LDH). The aim of the present review is different functions of nanomaterials as electrode modification materials, as well as applications of nanomaterials in electrochemical enzyme biosensors. For the sake of clarity, the last part of this review will specifically focus on our work evaluating the effect of nanomaterials on electrochemical biosensors using the electrochemical approach in view of the remarkable sensitivity observed.

## 2. Nanomaterials as Biosensor Modifiers of Glucose Oxidase (GOD) and Horseradish Peroxidase (HRP)

Owing to the clinical significance of blood glucose levels measurement, there are many sensitive and selective electrochemical biosensors for glucose fabricated by immobilizing glucose oxidase (GOD) in different matrices [20-23]. These biosensors show some practical advantages such as operational simplicity, low-cost fabrication and suitability for real-time detection, etc. [24,25]. Most of them are based on measuring the increase of the anodic current during the oxidation of hydrogen peroxide (H_2_O_2_) produced from the oxidation of glucose by dissolved oxygen in presence of GOD or the decrease of the cathodic current during the reduction of dissolved oxygen due to its consumption in the enzymatic reaction. Horseradish peroxidase (HRP) has long been a representative system for investigating the structures and properties of peroxidases, especially in understanding the biological behavior of the catalyzed oxidation of substrates by H_2_O_2_ [26,27]. H_2_O_2_ biosensors based on immobilizing HRP with nanomaterials have been developed, showing that nanomaterials can provide a desirable microenvironment to retain the bioactivity of HRP and display a good electrocatalytic response to H_2_O_2_.

### 2.1. GOD on ZrO_2_ Nanoparticles

The direct electron transfer between electrodes and glucose oxidase (GOD) immobilized in a matrix containing zirconium dioxide nanoparticles (ZrO_2_) is described in [28]. GOD was immobilized on a PG electrode using ZrO_2_ nanoparticles in the presence of either Pt-PLL or Pt-PVA, as well as in DMSO and DDAB aiming to achieve the fast electron transfer of GOD. The protein-nanoparticle assembly is stabilized by charged and uncharged compounds and the direct electron transfer is enhanced. The effects of different compositions on the electrochemical parameters, formal potential, surface loading, and constant heterogeneous electron transfer rate were characterized by cyclic voltammetry. The fastest electron transfer rate with the smallest deviation of the E° is obtained when GOD is immobilized with ZrO_2_ nanoparticles, colloidal platinum and poly-L-lysine (PLL).

**Figure 1.**
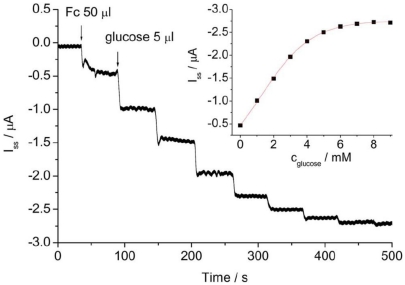
Amperommetric response of GOD/ZrO_2_/Pt-PLL/PG by successive addition of 5 μL glucose to 0.1 M pH 7.0 PBS containing 0.2 mM FcPF under stirring at 0.4 V. Inset: Calibration curve. Reprinted from reference [28] with permission from IEEE.

Electrochemical and spectroscopic measurements show that the GOD entrapped in ZrO_2_/Pt-PLL or ZrO_2_/Pt-PVA film retains its bioactivity efficiently and exhibits excellent electrocatalytic behavior towards glucose. No enzymatic activity of the immobilized GOD can be observed on ZrO_2_/DMSO and ZrO_2_/DDAB film. Figure 1 shows the amperometric response of GOD/ZrO_2_/Pt-PLL/PG at different concentrations of glucose in the presence of 0.2 mmol/mL FcPF_6_. The electrocatalytic anodic currents indicate the effective bioelectrocatalyzed oxidation of glucose at GOD/ZrO_2_/Pt-PLL/PG. As the electrocatalytic anodic current started at *E* = 0.23 V, the redox potential of the FcPF_6_, the latter mediated the electron transfer between the FAD redox center of the immobilized enzyme and the electrode. The electrocatalytic anodic currents increased as the concentration of glucose was elevated, and they leveled off at the glucose concentration of about 4 mmol/mL for GOD/ZrO_2_/Pt-PLL/PG.

### 2.2. HRP on TPSP-ZnO Nanostructure

**Figure 2.**
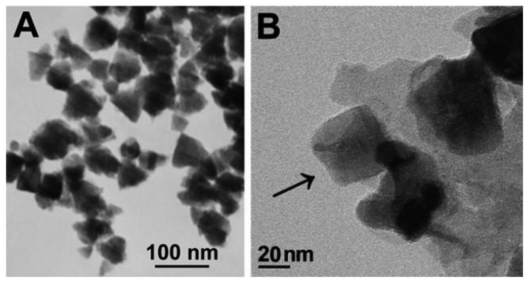
TEM images of the tetragonal pyramid-shaped ZnO particles. Panel (B) reveals the tetragonal pyramid shape of a ZnO particle (marked with an arrow). Reprinted from reference [29] with permission from The Royal Society of Chemistry.

A novel nano-sized tetragonal pyramid-shaped porous ZnO (TPSP-ZnO) structure (Figure 2) was, for the first time, prepared with a high morphological yield by a simple polyglycol-assisted wet chemical method. TPSP-ZnO can be used as an efficient matrix for immobilizing horseradish peroxidase (HRP) and applied to sense hydrogen peroxide (H_2_O_2_) [29].

**Figure 3.**
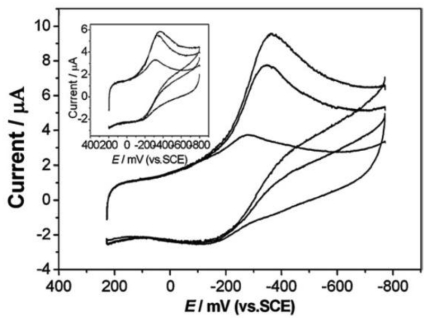
Cyclic voltammograms of HRP/TPSP-ZnO and HRP/spherical ZnO (inset) modified GCEs in 0.1 M PBS containing 0, 12 and 18 mM H_2_O_2_ (from bottom to top) at 100 mV s^-1^. Reprinted from reference [29] with permission from The Royal Society of Chemistry.

Upon the addition of H_2_O_2_, the shape of the cyclic voltammogram for the direct electron transfer of HRP changed dramatically, with an increase of reduction current and a decrease of the oxidation current (Figure 3), displaying an obvious electrocatalytic behavior of immobilized HRP to the reduction of H_2_O_2_. Interestingly, it had better biosensing properties than solid spherical ZnO nanoparticles, which might result from the larger specific surface area of TPSP-ZnO, causing a higher HRP loading, and the tetragonal pyramid-shaped porous nanostructure having high fraction of surface atoms located on the corners and edges, resulting in an improved catalytic activity.

### 2.3. A GOD-HRP Bienzyme Channeling Glucose Sensor in SBA-15 Mesopores

**Figure 4.**
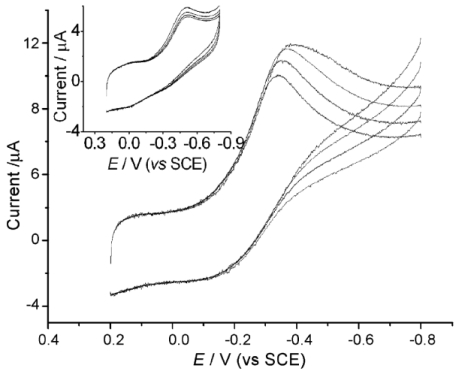
Cyclic voltammograms of HRP–GOD/SBA-15 modified GCE in 0.1 M 7.0 PBS containing 0, 1.0, 2.0 and 3.0 mM glucose (from bottom to top) at 0.10 V/s. Inset: cyclic voltammograms of HRP–GOD modified GCE in 0.1 M 7.0 PBS containing 0, 1.0, 2.0 and 3.0 mM glucose (from bottom to top) at 0.10 V/s. Reprinted from reference [30] with permission from Elsevier Science B.V.

A novel bienzyme-channeling sensor was constructed by entrapping glucose oxidase (GOD) and horseradish peroxidase (HRP) in the mesopores of well-ordered hexagonal mesoporous silica structures (SBA-15) by simply immersing SBA-15 in the enzyme solution [30]. The SBA-15 mesoporous materials accelerated the electron transfer between the entrapped HRP and electrode (Figure 4). Both HRP and GOD retained their catalytic activities in the bienzyme-entrapped SBA-15 film. In presence of glucose the enzymatic reaction of the GOD-glucose-dissolved oxygen system generated hydrogen peroxide in the bienzyme-entrapped mesopores, which was immediately reduced at −0.40 V by an electrocatalytic reaction with the HRP entrapped in the same mesopore to lead to a sensitive and fast amperometric response. Thus bienzyme channeling could be used for the detection of glucose with excellent performance without the addition of any mediator.

**Figure 5.**
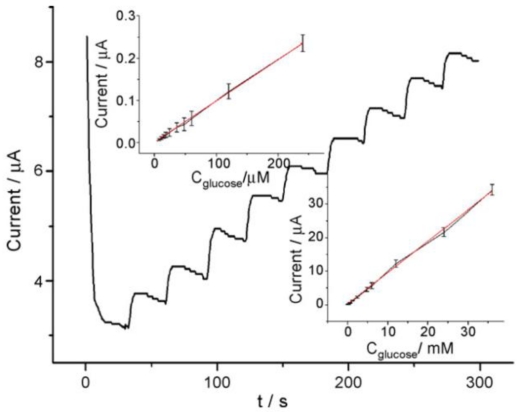
Amperometric response of GOD–HRP/SBA-15 modified GCE upon successive additions of 5 μL 0.4M glucose to 5.0 mL 0.1 M pH 7.0 PBS at −0.40 V. Insets (A) and (B): linear calibration curve of glucose sensor with five reduplicate measurements. Reprinted from reference [30] with permission from Elsevier Science B.V.

Optimization of the experimental parameters was performed with regard to pH, operating potential and temperature. The detection limit was down to 2.7 × 10^−7^ M with a very wide linear range from 3.0 × 10^−6^ to 3.4 × 10^−2^ M (Figure 5). The constructed bienzyme channeling provided a strategy for amperometric detection of oxidase substrates by co-entrapping the corresponding oxidase and HRP in the mesoporous materials.

## 3. Nanomaterials as Modification Biosensors of Cytochrome P450 (CYP2B6) and Hemoglobin (Hb)

Cytochrome P450, family 2, subfamily B, polypeptide 6 (CYP2B6) expressed in human liver microsomes is a heme enzyme which can catalyze a various of xenobiotics, including carcinogens, nicotine, styrene, tamoxifen, and antipyrine [31-33]. Several studies have confirmed that certain drugs, such as RP73401, 3-cyano-7-ethoxycoumarin, imipramine, 7-ethoxycoumarin, lidocaine, cyclophosphamide (CPA) and bupropion (BUP), are also metabolized predominantly by CYP2B6 in the human liver [34-40]. The research exploring the use of cytochrome P450 in bioreactors or biosensors suggested that an electrode could be used to substitute for reductases in the enzyme-based biological electron delivery and transport systems [41-43]. Hence, CYP2B6, as well as hemoglobin (Hb, another heme enzyme), were chosen for studying the electrochemical activity and catalytic reactions of the immobilized protein and the preparation of relatively sensitive biosensors.

### 3.1. CYP2B6 on ZrO_2_ Nanoparticles

**Figure 6.**
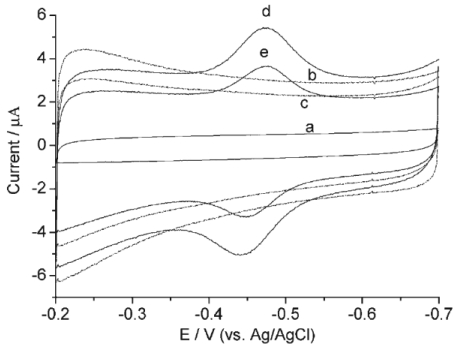
Cyclic voltammograms of a) GCE, b) Pretreated GCE, c) ZrO_2_/PLL/GCE, d) CYP2B6/ZrO_2_/Pt-PLL/GCE, and e) CYP2B6/GCE in 0.1 M pH 7.4 PBS at 100 mV/s. Reprinted from reference [44] with permission from Wiley-VCH.

The direct electrochemical and electrocatalytic behavior of the immobilized cytochrome P450 2B6 (CYP2B6) on zirconium dioxide nanoparticles (ZrO_2_) was investigated [44]. The film of nano-structured ZrO_2_ that incorporated cytochrome P450 2B6 (CYP2B6) with colloidal platinum, which was stabilized by poly-l-lysine (Pt-PLL), was prepared on glassy carbon electrodes. Figure 6 shows the cyclic voltammograms of different electrodes in 0.1 M pH 7.4 oxygen-free PBS at 100 mV/s. When CYP2B6 was cast directly on the electrode surface, the CYP/GCE also showed the response of CYP2B6, but the response was 1.6 times smaller than that of CYP/ZrO_2_/Pt-PLL/GCE. Thus the adsorption of CYP2B6 on ZrO_2_ nanoparticles played an important role in facilitating the electron exchange between the electroactive center of CYP2B6 and GCE. The *E*′ of CYP2B6 in theZrO_2_/Pt-PLL-film, estimated to be the midpoint of anodic andcathodic peak potentials in the scan rate range, was −(0.449 ± 0.004) V at pH 7.4.

In air-saturated solutions, an increased bioelectrocatalytic reduction current could be obtained with the CYP2B6-modified electrode with the addition of drugs, such as lidocaine. This leads to the construction of disposable biosensors for drugs by utilizing the electrochemical activity and catalytic reactions of the immobilized CYP2B6.

### 3.2. CYP2B6 on Au–chitosan/GCE Film

**Figure 7.**
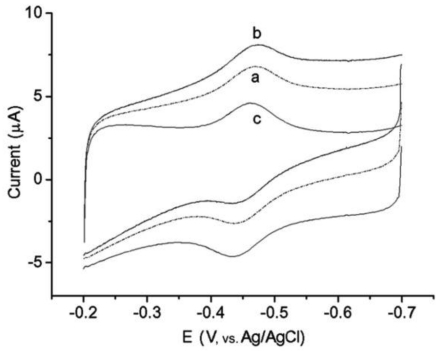
Cyclic voltammograms of a CYP2B6/Au–chitosan modified GCE in PBS (0.1 M, pH 7.4) that was air saturated (a), air saturated with the addition of 0.2 mM BUP (b), and nitrogen saturated (c). Scan rate: 100 mV/s. Reprinted from reference [45] with permission from Elsevier Science B.V.

An improved system to study the two-electron delivery reaction pathway of CYP2B6 *in vitro* has been described. In particular, a biocompatible film containing colloidal gold nanoparticles and chitosan was used to encapsulate CYP2B6 on an electrode [45]. The electrocatalytic behaviors of CYP2B6 toward common drugs in the absence of NADHP–cytochrome P450 reductase as electron donor were studied. In an anaerobic solution, direct and reversible electron transfer between the electroactive heme center of CYP2B6 and the electrode was observed with a formal potential of −0.454 ± 0.006 V at pH 7.4 (Figure 7).

In an air-saturated solution, an increase in the bioelectrocatalytic reduction current was observed after drug [bupropion (BUP), cyclophosphamide (CPA) and lidocaine] addition. The bioelectrocatalytic products were analyzed using high-performance liquid chromatography (HPLC) and electrospray ionization–mass spectrometry (ESI–MS). Both results confirmed that C-hydroxylation and heteroatom release were the main pathways for CYP2B6-mediated drug oxidation, similar to what occurred *in vivo*. The use of immobilized proteins in nanoparticle-containing films in drug biosensing was also demonstrated.

### 3.3. Hb on PUE/MWNT/PG Electrode

In this study, we investigated the direct electron-transfer reactivity of immobilized hemoglobin (Hb) on a polyurethane elastomer (PUE) film for biosensor design [46]. The PUE film synthesized by an additional polymerization possesses good biocompatibility, uniformity, and conformability and is ready for protein immobilization.

Electrochemical and spectroscopic measurements show that the presence of multiwalled carbon nanotubes (MWNTs) increased the protein-PUE interaction, varied polymer morphology, improved the permeability and the conductivity of the PUE film, and thus facilitated the direct electron transfer between the immobilized Hb and the conductivity surface through the conducting tunnels of MWNTs. The immobilized Hb maintains its bioactivities and displays an excellent electrochemical behavior with a formal potential of −(334 ± 7) mV.

**Figure 8.**
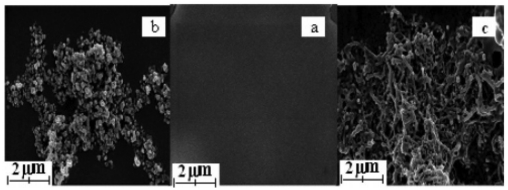
Scanning electron micrographs of (a) PUE, (b) Hb/PUE, and (c) Hb/PUE/MWNT films on a glass slide. Reprinted from reference [46] with permission from The American Chemical Society.

Our SEM measurement shows that in the presence of MWNTs the film displays a chemically clean, unique three-dimensional netlike porous structure (Figure 8). This uniform open porous structure provides a significant increase in the effectiveness of the electrode surface for Hb loading, decreases the reorganizational energy for electron transfer, and allows the electroactive probe to easily diffuse through the films. This results in a good electrochemical response from Fe(CN)_6_^3−/4−^ and a direct electrochemical response of the immobilized Hb.

**Figure 9.**
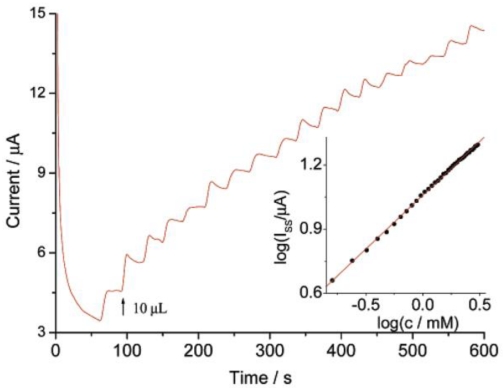
Amperometric response of the Hb/PUE/MWNT/PG electrode by successive addition of 10 μL of 40 mM NaNO_2_ to 5 mL of air-free 0.2 M HAc-NaAc, pH 4.0, under stirring at −0.7 V. Inset: Linear calibration curve. Reprinted from reference [46] with permission from The American Chemical Society.

The addition of NaNO_2_ leads to an increase of the electrocatalytic reduction current of nitrite at −0.7 V. This allows us to develop a nitrite sensor with a linear response range from 0.08 to 3.6 mM (Figure 9). The proposed method opens a way to develop biosensors by using nanostructured materials mixed with low electrical conductivity matrixes.

## 4. Evaluation the Effect of Nanomaterials on the Enzyme Activity

We established a new method for evaluating the toxic effect of nanomaterials on the enzyme activity using the electrochemical approach. The differential-pulse polarography (DPP) technique was applied to study the effects of Al^3+^ ion on the glutamate dehydrogenase (GDH) activity in the catalytical reaction of α-KG + NADH + NH_4_^+^ ⇔ l-Glu + NAD+ + H_2_O by monitoring the DPP reduction current of NAD^+^ [47].

**Figure 10.**
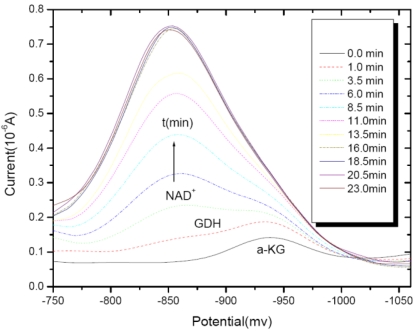
Differential-pulse polarography of GDH enzyme system; in a 0.1 M pH = 6.5 Tris-HCl buffer solution; 0.002 M α-KG; 0.0002 M NADH; 0.01 M NH_4_Cl; 4 μL GDH; Scan rate: 5 mV/s. Reprinted from reference [47] with permission from MDPI.

Figure 10 reports the differential-pulse polarography in a buffer solution of 100 mM Tris-HCl (pH 6.5) +2 mM α-KG +10 mM NH_4_Cl and 0.2 mM NADH with a final volume of 20 mL. It clearly shows that a well-defined higher differential-pulse peak potential of the NAD^+^ reduction current at −0.86 V. Meanwhile, a rather weaker peak was at −0.97 V for α-KG, which was also agreed with our previous finding for α-KG in case of the different potentials caused by different pH values [48]. With the addition of 4 μL of GDH enzyme to the assay solution, the peak current of NAD^+^ increased whereas that of α-KG decreased as the reaction time evolved, which means that the GDH catalytical reaction started and finally reached reversible equilibrium. It is, therefore, clearly revealed that the differential pulse polarography technique could accurately determine the GDH activity in an enzymatic catalytical reaction.

**Figure 11.**
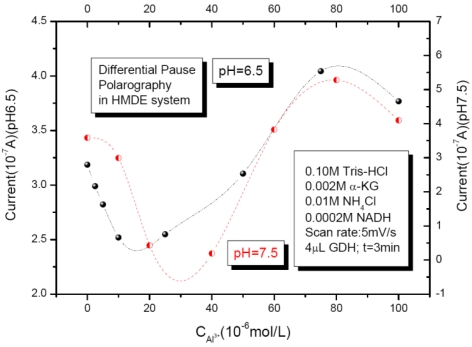
DPP peak currents as a function of Al (III) concentration; in 0.1 M pH = 6.5 and 7.5 Tris-HCl buffer solutions; 0.002 M α-KG; 0.01 M NH_4_Cl; 0.0002 M NADH; 4 μL GDH; measurement time 3 min; Scan rate: 5 mV/s. Reprinted from reference [47] with permission from MDPI.

In the presence of Al (III) in the above enzyme system, the peak currents of NAD+, which were converted from NADH by GDH and measured at the same reaction time (3 min) are shown in Figure 11. At the plant and animal physiologically relevant pH values (pH = 6.5 and 7.5), the GDH enzyme activities were strongly dependent on the concentrations of the metal ion in the assay mixture solutions. In the lower Al (III) concentration solutions (<30 μM), inhibitory effects were shown, which are in accordance with the recent biological findings. With the increase of Al (III) concentrations (30∼80 μM), the enzyme GDH activities were activated. However, once the concentration of Al (III) arrived to near 0.1 mM level (>80 μM), the inhibition effects of Al (III) were shown again.

The cyclic voltammetry of NAD^+^ and NAD^+^-GDH in the presence of Al (III) can help to explain some biological phenomena. According to the differential-pulse polarography and cyclic voltammetry experiments, the present research confirmed that the electrochemical technique is a convenient and reliable sensor for accurate determination of enzyme activity in biological and environmental samples.

Recently, the effects of nanometer-sized tridecameric aluminum polycation (nano-Al_13_) on lactate dehydrogenase (LDH) activity have been investigated using differential pulse voltammetry (DPV) at the molecular level. The effects of nano-Al_13_ on LDH activity at different temperature and pH values were investigated. The results showed that these factors had quite different influences. The characteristic parameters of Michaelis constant *K*_m_ and maximum velocity V_max_ were evaluated by monitoring the DPV reduction current of NAD^+^ (β-nicotinamide adenine dinucleotide) and compared with the other known inhibitors of LDH. Considering the significance of LDH in the area of medicine, biology, and biomarkers, this method can be conveniently applied to evaluate the effects of nanomaterials on environment by constructing the small and portable biosensors. It could be anticipated to satisfy many requirements for analyzing different biological systems and might provide a useful reference for assessing the toxic and biological effects of nanomaterials by detecting enzyme activity [49].

## 5. Concluding Remarks

We have featured some recent advances in nanomaterial applications in electrochemical enzyme biosensors. Nanomaterials offer superior ways for the maintenance of enzyme activity and the improvement of the sensitivity of biosensors. With the development of nanoscience and nanotechnology, the combination of various nm scale nanomaterials with enzymes could lead to the development of multi-functional nano-assembly systems with simultaneous novel electronic properties. Such coupling of high sensitivity and stability capabilities allows electrochemical biosensors to rival the most advanced electrochemical and optical protocols in bioassays.

Considering the significance of enzymes in the area of medicine, biology, and biomarkers, our recent works in electrochemical biosensors can be conveniently applied to evaluate the effects of nanomaterials on the environment. It could be anticipated to satisfy many requirements for analyzing different biological systems and might provide a useful reference for assessing the toxic and biological effects of nanomaterials by detecting enzyme activity. The electrochemical method for the investigation of metal and semiconductor nanomaterials illustrates the extraordinary sensitivity achievable by this technique. The integration of the technologies will, without doubt, bring significant input to ultra-sensitive biosensors relevant to diagnostics, therapy and controlled drug delivery of interest for human health.
